# 3D Printed Strontium and Zinc Doped Hydroxyapatite Loaded PEEK for Craniomaxillofacial Implants

**DOI:** 10.3390/polym14071376

**Published:** 2022-03-28

**Authors:** Faisal Manzoor, Atefeh Golbang, Dorian Dixon, Elena Mancuso, Usaid Azhar, Ioannis Manolakis, Daniel Crawford, Alistair McIlhagger, Eileen Harkin-Jones

**Affiliations:** 1 Department of Mechanical Engineering, School of Engineering, Ulster University, Shore Road, Newtownabbey BT37 0QB, UK; a.mcilhagger@ulster.ac.uk (A.M.); e.harkin-jones@ulster.ac.uk (E.H.-J.); 2 Nanotechnology and Integrated Bio-Engineering Centre (NIBEC), Ulster University, Shore Road, Newtownabbey BT37 0QB, UK; d.dixon@ulster.ac.uk (D.D.); e.mancuso@ulster.ac.uk (E.M.); 3 Precision Engineering, Materials & Manufacturing (PEM) Research Centre, Institute of Technology Sligo, Ash Lane, F91 YW50 Sligo, Ireland; usaid.azhara@mail.itsligo.ie (U.A.); manolakis.ioannis@itsligo.ie (I.M.); 4 Department of Life Sciences, Institute of Technology Sligo, Ash Lane, F91 YW50 Sligo, Ireland; 5 Axial 3D, Alexander House, 17a Ormeau Ave, Belfast BT2 8HD, UK; d.crawford@axial3d.com

**Keywords:** 3D printing, fused deposition modelling, PEEK, bioactive composites, doped hydroxyapatite

## Abstract

In this study, Strontium (Sr) and Zinc (Zn) doped-HA nanoparticles were synthesized and incorporated into polyetheretherketone (PEEK) up to 30 wt.% and processed by a novel approach i.e., fused deposition modelling (FDM) 3D printing for the production of patient specific cranial implants with improved bioactivity and the required mechanical performance. Filaments were produced via extrusion and subsequently 3D-printed using FDM. To further improve the bioactivity of the 3D-printed parts, the samples were dip-coated in polyethylene glycol-DOPA (PEG-DOPA) solution. The printing quality was influenced by filler loading, but was not significantly influenced by the nature of doped-HA. Hence, the printing conditions were optimized for each sample. Micro-CT and Scanning Electron Microscopy (SEM) showed a uniform distribution of bioceramic particles in PEEK. Although agglomeration of particles increased with increase in filler loadings. Differential Scanning Calorimetry (DSC) showed that the melting point and crystallinity of PEEK increased with an increase in doped-HA loading from 343 °C to 355 °C and 27.7% to 34.6%, respectively. Apatite formation was confirmed on the 3D-printed samples after immersion in simulated body fluid (SBF) for 7, 14 and 28 days via SEM, X-ray diffraction (XRD) and Fourier Transform Infrared Spectroscopy (FTIR). The tensile strength and impact strength decreased from 75 MPa to 51 MPa and 14 kJ/m^2^ to 4 kJ/m^2^, respectively, while Young’s modulus increased with increasing doped-HA content from 2.8 GPa to 4.2 GPa. However, the tensile strengths of composites remained in the range of human cortical bone i.e., ≥50 MPa. In addition, there was a slight increase in mechanical strength after 28 days immersion which was attributed to apatite formation. Water contact angle showed that the hydrophilicity of the samples improved after coating the 3D-printed samples with PEG-DOPA. Hence, based on the results, the 3D-printed PEEK nanocomposites with 20 wt.% doped-HA is selected as the best candidate for the 3D-printing of craniomaxillofacial implants.

## 1. Introduction

Fused deposition modelling (FDM) also referred to as fused filament fabrication (FFF), has revolutionized the healthcare sector by enabling the 3D printing of personalized implants and medical devices with high accuracy, combined with low manufacturing costs, material waste and environmental impact [1,2,3]. FDM of biomedical implants has become very attractive particularly for the repair of cranio-maxillofacial defects [4,5]. These defects arise due to traumas, infections, tumours and/or congenital deformities and are highly challenging for surgeons to reconstruct [6,7]. When the bone defect is greater than the critical size defect, then autologous bone graft is the first choice due to the immune similarity [8]. However, bone graft resorption, infection and bone geometry/structure restrict the application of autologous bone grafts [9]. Currently, the most popular synthetic materials for repairing such defects are metals (i.e., titanium and its alloys) due to their corrosion resistance, excellent biocompatibility, and high mechanical properties [10]. However, in recent years, metal implants are being replaced by polymeric implants for bone repair due to the limitations associated with metals, such as stress-shielding, high specific weight, complexities during radiography and the release of harmful metal ions into the blood stream [11,12].

An ideal biomaterial for implant use should possess adequate mechanical properties (i.e., similar to that of the original body tissue) as well as a bioactive surface for integration. Poly(ether ether ketone) (PEEK) is an ideal candidate material for cranial and maxillofacial bone repair [13,14,15]. It is a semicrystalline thermoplastic polymer with elastic modulus (3–4 GPa) in the range of human cortical bone. It is also biocompatible, radiolucent, and stable after sterilization with gamma irradiation [16]. However, PEEK is hydrophobic and therefore largely bioinert, which limits its adhesion to surrounding tissues [17]; however, this can be altered either by the incorporation of bioactive fillers such as hydroxyapatite (HA) or bioactive glass [18,19,20,21], or by coating with a suitable material [22,23,24]. HA is a synthetic bioceramic material which chemically resembles the inorganic phase of human bone [25]. Additionally, the biological performance of HA can be enhanced by doping it with other elements, such as strontium (Sr), silicon (Si), magnesium (Mg), and zinc (Zn), constituents of human bone in various amounts, depending on the type of tissue (i.e., bone, dentin, enamel). These doped elements play an important role in bone regeneration and biomineralization [26]. For example, Sr promotes bone growth and inhibits bone resorption [27], Si enhances the bioactivity of HA and bone regeneration in vivo [28], Mg enhances HA resorption and is a key factor in the activity of several enzymes [29], while Zn acts as a co-factor for several enzymes, promotes bone growth and has anti-bacterial properties [30]. Feng et al., reported multimaterial scaffolds of PEEK with biodegradable poly (L-lactide) (PLLA) and β-tricalcium phosphate (β-TCP), in which PLLA degraded and provided the enhanced bioactivity of multimaterial scaffolds [31]. Additionally, PEEK has been coated with various materials to improve its hydrophilicity [32,33,34]. 1-3,4-dihydroxyphenylamine (DOPA) has exceptional adhesive characteristics and it produces a biocompatible hydrogel when conjugated with polyethylene glycol (PEG) [35]. The structure, synthesis and characterisation of the compound used (PEG1000-DOPA) for coating the 3D-printed PEEK and PEEK nanocomposites in this study have been described in detail elsewhere [20]. In brief, PEG1000-DOPA is a linear homobifunctional oligo(ethylene glycol) with Mn = 1000 Da and catechol-modified chain-ends, from the reaction of a hydroxy-terminated PEG1000 precursor with L-DOPA.

One of the objectives of this study is to evaluate the printability and bioactivity of PEEK/doped-HA (loaded with Sr and Zn) nanocomposites for cranial implants. Although FDM offers many benefits for the production of customized biomedical implants, one of the major downsides of this manufacturing method is that the mechanical properties of the printed parts are commonly lower than those of parts manufactured using traditional methods, such as injection moulding [36]. Thus, another objective of this study is to characterize the 3D-printed parts via mechanical testing to see if they are in the acceptable range for use as cranial implants. In a study about PEEK/doped HA composites that was conducted by Wang et al., PEEK composites with 40 wt.% nano-flourohydroxyapatite (nano-FHA) were prepared by compression moulding and then surface treated using TiO_2_ blasting to study the effect of surface roughness. The authors reported excellent bioactivity, antibacterial properties, osseointegration and bone-implant contact which was attributed to the synergistic effect of surface roughness and nano-FHA particles [37]. Recently, several studies have explored the 3D printing of PEEK and PEEK/HA composites via FDM [19,38,39,40,41]. To the author’s knowledge no previous publication has investigated FDM printed PEEK composites containing doped-HA, although there have been studies on the processing of PEEK/doped-HA composites produced via conventional technologies. Wong et al. prepared PEEK/SrHA composites via compression moulding with varying amounts of SrHA ranging from 0 to 30 vol.%. They reported that as the vol.% of SrHA increased, the bending strength of the composite decreased while the bending modulus increased. A PEEK sample with 25 vol.% of SrHA showed enhanced apatite formation ability in SBF and enhanced MG-63 cell attachment ability as compared to 25 vol.% of pure HA with PEEK [42]. In another study reported by Faith, electrostatically bonded PEEK composites were fabricated by the incorporation of 5 and 10 vol.% of SrHA via cold pressing and then characterized; it was reported that the compression strength and hardness of disc shaped samples increased while the modulus decreased. The samples showed apatite layer formation on the surface after incubation for 14 days in SBF [43].

The 3D-printing of PEEK and its composites via FDM is challenging due to its high melting point and high viscosity. In addition, the semi-crystalline nature of PEEK causes high residual stresses, resulting in shrinkage, warpage and thermal cracks in the final developed structure [44,45]. Optimization of the printing conditions is therefore required to improve print quality and final properties of PEEK 3D-printed parts produced via fused filament methods [19,39,40,45,46,47,48]. However, there is still a lack of literature regarding the processing of PEEK composites with doped HA by 3D printing.

This study aims to evaluate the 3D-printability and bioactivity of Sr and Zn doped HA/PEEK nanocomposites with 0, 10, 20 and 30 wt.% filler content to analyse their mechanical performance and bioactive potential. PEEK nanocomposite filaments were prepared via extrusion and then 3D printed via FDM under optimized printing conditions to achieve good print quality and desirable mechanical properties for cranial implants. The morphology, thermal and mechanical properties, hydrophilicity as well as the bioactivity of the samples were studied. The effect of a PEG_1000_-DOPA coating on the surface hydrophilicity of the samples containing 20 wt.% bioceramic particles was also studied with the objective to improve bioactivity. Furthermore, the effect of SBF immersion on the mechanical properties of 3D-printed samples up to 28 days was investigated for the first time.

## 2. Materials and Methods

All the reagents for the synthesis of SrHA and ZnHA were of analytical grade and purchased from Sigma Aldrich (Gillingham, Dorset, UK). Strontium doped and zinc doped hydroxyapatites (5% *w*/*w*) were synthesized via a wet chemical precipitation method by adding phosphoric acid (H_3_PO_4_) dropwise into a solution containing calcium hydroxide and strontium nitrate (or zinc nitrate for ZnHA), and as previously reported [49]. The resulting doped HA powders were sintered at 900 °C by heating at 10 °C/min from room temperature and held for 4 h at 900 °C in air and then ground and sieved through a 180-micron sieve to remove any larger particles which could block the nozzle during 3D printing. Then, SrHA and ZnHA powders were physically mixed with commercially available PEEK powder from Evonik (VESTAKEEP^®^ 2000UFP, GmbH Germany) at loading levels of 0, 10, 20 and 30 wt.%. The mixtures were then dried at 110 °C overnight before being extruded into continuous 1.75 ± 0.05 mm diameter filaments via a desktop extruder (3devo composer 450, Utrecht, The Netherlands). The filaments were labelled as PEEK, PEEK/10SrHA, PEEK/20SrHA, PEEK/30SrHA, PEEK/10ZnHA, PEEK/20ZnHA and PEEK/30ZnHA.

### 2.1. Optimization of Filament Extrusion and 3D Printing

The filaments with a diameter of 1.75 ± 0.05 mm were obtained after optimization of the various extrusion process parameters such as powder feeding rate, extrusion temperature, screw speed, cooling fan speed, puller wheel speed and nozzle diameter. The final optimised conditions for each material are summarized in Table 1. The viscosity of the molten material depends upon temperature and the filler content, thus fine temperature control is critical. The fed powder passed through four different heating zones (Z_1_, Z_2_, Z_3_, Z_4_) and the temperature of each zone was controlled separately. For pure PEEK, the temperature of zone 1 (Z_1_) was set to 355 °C which was slightly above the melting point of PEEK and then the temperature was gradually increased in the later zones to 365 °C, 375 °C and 390 °C. Similarly, the extrusion temperatures were optimised for PEEK nanocomposites and have been summarized in Table 1. The desired diameter of filaments was obtained after carefully adjusting the cooling rate and puller wheels speed by keeping the screw speed and feeding rate constant, depending on the amount of bioceramic particles. The non-uniform and uniform diameter filaments obtained during optimization have been reported in Figures S1 and S2 (please see the supplementary information). The summary of optimized extrusion parameters for producing each nanocomposite filament are reported in Table 1.

The most critical parameters for optimizing the 3D printing of the PEEK/doped HA samples were found to be layer thickness, printing speed, print bed temperature, nozzle temperature and chamber temperature (SpiderBot 4.0 HT, Lugny, France). Initially, lower temperatures were used to print the samples. For example, for the 3D printing of pure PEEK, a 360 °C nozzle temperature (about 20 °C higher than the PEEK melting point, as presented in Section 3.1.2), 120 °C bed and 60 °C chamber temperatures were applied. However, these prints failed due to warpage and poor adhesion of the first layer. Hence, the samples were prepared successfully with good print quality by gradually increasing the temperatures to 390 °C nozzle temperature, 150 °C bed temperature (above the glass transition temperature of PEEK) and 75 °C chamber temperature. Similarly, the temperatures for the PEEK nanocomposites were optimized. The pictures of 3D printed samples for impact testing during the optimization have been reported in Figure S3 (please see the supplementary information). In addition, different printing speeds (20, 30, 40 and 50 mm/s) were tested to further optimize the 3D printing process. The samples prepared with a printing speed of 20 mm/s showed poor adhesion between the layers which is likely due to the solidification of previously printed layers and incomplete fusion between layers. Additionally, the higher printing speeds of 40 and 50 mm/s exhibited poor surface quality with small pores and gaps on the surface which would reduce the mechanical properties. Hence, 30 mm/s printing speed was found to be optimum for printing samples. Similarly, the layer thickness varied between 0.4 mm and 0.2 mm under the optimized printing temperatures and printing speed. Based on the tensile testing data (following ISO 527-2 standard), a layer thickness of 0.2 mm showed improved tensile strength compared to a layer thickness of 0.4 mm. A layer thickness of 0.2 mm resulted in a tensile strength in a similar range to that of cortical bone (~50 MPa) [50] and was therefore chosen as the optimized parameter. Table 2 shows a summary of the optimized printing conditions for the PEEK and PEEK/SrHA samples with different filler loadings. The same process was applied for PEEK/ZnHA nanocomposites and similar results were obtained. Hence, the optimized printing conditions for PEEK/ZnHA are the same as those reported for PEEK/SrHA nanocomposites at each filler loading. The final optimized printing conditions for each sample are given in Table 2.

### 2.2. Physicochemical Characterization of Extruded Filaments and 3D Printed Samples

Prior to 3D printing, the distribution of the bioceramic particles in the filaments was investigated using micro-computed tomography (µ-CT; Microtomograph SkyScan 1275 Bruker, Billerica, MA, USA) with source voltage 40 kV, source current 25 µA and pixel size 10 µm. The top view of the surface morphology and the distribution of bioceramic particles in the 3D printed samples were observed through field emission scanning electron microscopy (FESEM; HITACHI SU5000, Tokyo, Japan). The samples were gold sputter coated prior to analysis and observed under high vacuum at 10 kV. The crystalline phases of the 3D-printed composite samples were identified by X-ray diffraction (XRD; PANanalytical X’Pert Pro, Malvern Panalytical, Malvern, UK) in 2θ range 10° to 80° with Cu Kα radiation (λ = 1.54 Å). The thermal properties (glass transition, melting and recrystallization temperatures) of the materials were analysed using Differential Scanning Calorimetry (DSC; Q100 TA Instruments, NJ, USA). The tests were carried out in accordance with ISO 11357 at a heating rate of 10 °C/min under flowing nitrogen. The samples were heated from 25 °C to 400 °C with a heat-cool-heat cycle. The crystallization temperatures were measured in the first cooling cycle whereas the melting temperatures were recorded during the second heating cycle to remove the influence of thermal history. The degree of crystallinity was calculated using the formula in Equation (1):

(1) $$Xcw\;\left(\%\right)=\frac{\mathrm{Hm}}{\mathrm{Wf}\;*\;\mathrm{Hc}}\;*\;100$$

Hm is the melting enthalpy acquired from the DSC scan, Wf is the weight fraction of PEEK polymer in nanocomposites and Hc is the melting enthalpy of fully crystallized PEEK (130 J/g) [51].

Prior to PEG_1000_-DOPA coating fabrication, the samples were wiped with acetone to remove any surface contamination. PEG_1000_-DOPA was dissolved in Tris HCl aqueous buffer (pH 8.5) at a concentration of 2 mg/mL. The samples were then immediately immersed in this solution for 18 h at room temperature, using a laboratory dip-coater (Ossila, Sheffield, UK). The samples were clamped and immersed vertically in the PEG_1000_-DOPA solution using the dip-coater arm. After completing the coating process, the samples were removed from the solution, rinsed with distilled water to remove any unbound PEG_1000_-DOPA molecules and dried under nitrogen flow for 2–3 min.

Static water contact angle was measured to assess the samples hydrophilicity using a Surftens Basic contact angle instrument (OEG GmbH, Hessisch Oldendorf, Germany). In total, five measurements were taken at different sites. The samples were placed on the observation stage and a droplet of deionised water (typically between 0.5 and 1.0 μL) was manually released onto the surface, using a Luer-lock glass syringe. The contact angle value was calculated from the obtained images using both sides of the droplet with Surftens Automatic 4.7 software, 5 droplets were measured for each sample type and the results are expressed as the average value ± standard deviation.

The tensile and impact properties of the 3D-printed samples were determined according to ISO 527-2 type 5A (5 dog-bone shaped samples, overall length ≥ 75 mm, gauge length = 20.0 ± 0.5 mm, thickness = 2.0 ± 0.2 mm) and ISO 180:2000 (5 rectangular samples, length = 80 ± 2 mm, width = 10.0 ± 0.2 mm, thickness = 4.0 ± 0.2 mm), respectively. The pictures of 3D printed samples for tensile testing have been provided in Figure S4 (please see the supplementary information). Tensile testing was performed on each type of sample (5) by an INSTRON mechanical testing machine (Model 5500R, Instron Limited, Norwood, UK) at a cross head speed of 5 mm/min using a 5 kN load cell. Impact testing was carried out on each type of unnotched samples (5 in total) using an Izod impact testing machine with a 0.5 kg hammer weight and falling speed of 20 mm/s.

The bioactivity and the effect of the apatite layer on the mechanical performance of the 3D-printed samples were evaluated via a simulated body fluid (SBF) immersion test and prepared according to Kokubo’s protocol [52]. The disk samples (3 in total) for bioactivity and the dog-bone samples (5 in total) of each group for mechanical evaluation were immersed in the SBF for 0, 7, 14 and 28 days. The SBF solution was replenished with fresh solution after 48 h to avoid dynamic equilibrium. The apatite layer formation on the surface of the samples was characterized via SEM. Fourier Transform Infrared Spectroscopy (FTIR) was performed using Thermoscientific (model iD5, Altrincham, UK) instrument with a scan range of 550 cm^−1^ to 4000 cm^−1^ and a resolution of 8 cm^−1^ in Attenuated Total Reflection (ATR) mode.

In order to check the repeatability of the results, the tensile strength, Young’s Modulus, water contact angle and impact strength are reported as the mean ± standard deviation (SD) (for 5 test specimens).

## 3. Results and Discussion

### 3.1. Filament Characterization

**Figure 1:**
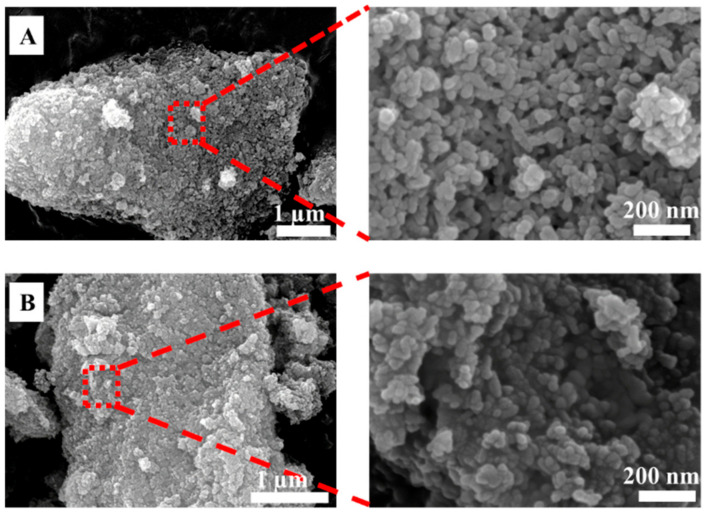
SEM analysis of doped-HA powders synthesized by the wet chemical precipitation method and sintered at 900 °C, (**A**) Strontium doped hydroxyapatite (SrHA) and (**B**) Zinc doped hydroxyapatite (ZnHA).

Prior to filament preparation, the synthesized doped-HA powders were characterized for their particle sizes and morphologies via SEM, as shown in Figure 1. The synthesized powders of doped-HA showed that the particle sizes were in the range of 60 to 80 nm and 40 to 60 nm for SrHA and ZnHA, respectively. The particles were round and partly fused together due to the sintering at 900 °C. Those powders were then uniformly mixed with PEEK powder at different concentrations (0 to 30 wt.%) to avoid agglomeration which could affect the mechanical and biological properties.

#### 3.1.1. Micro-CT Analysis of Filaments

**Figure 2:**
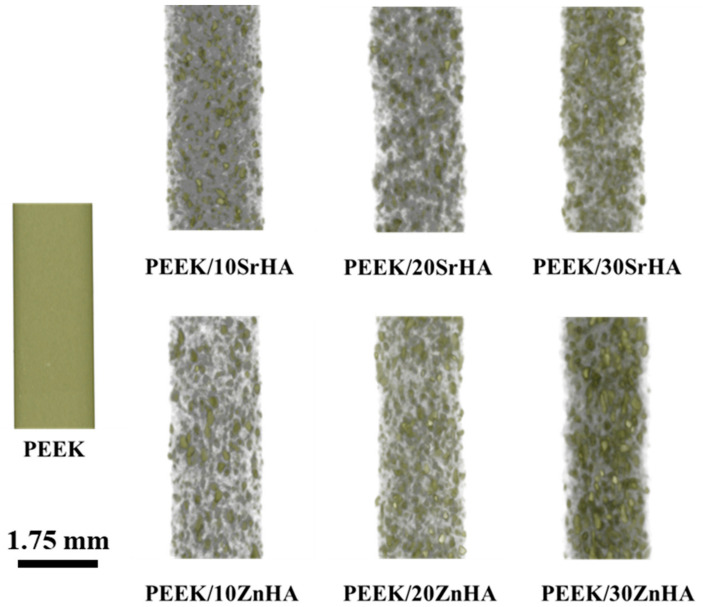
Micro-CT of PEEK filament and PEEK/doped-hydroxyapatite filaments produced via extrusion, showing a uniform distribution of bioceramic particles, the diameter of each filament is 1.75 ± 0.05 mm.

The distribution of bioceramic particles in filaments was observed by micro-CT analysis as shown in Figure 2. The small grey dots represent the bioceramic particles. The concentration of the grey dots increases as the bioceramic percentage increases from 10 to 30 wt.%. It is evident from Figure 2 that the distribution of the particles is uniform throughout the samples, without any significant agglomeration.

#### 3.1.2. Thermal Analysis of Filaments

**Figure 3:**
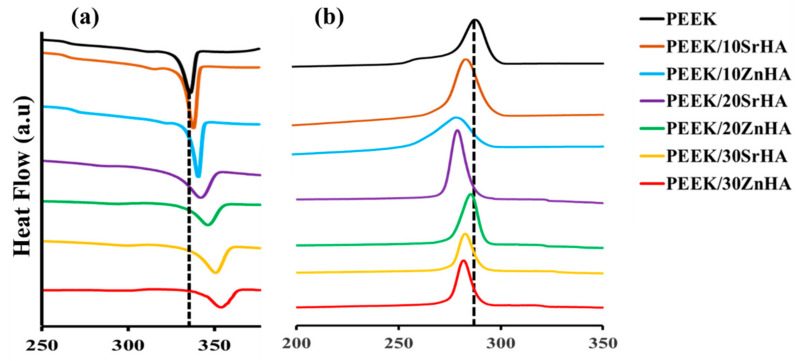
Thermal properties of filaments, (**a**) represents the melting temperature (T_m_) and (**b**) represents the crystallization temperature (T_c_) of PEEK with Strontium doped hydroxyapatite (SrHA) and Zinc doped hydroxyapatite (ZnHA).

Figure 3a,b show the melting temperatures (T_m_) and crystallization temperatures (T_c_) of the PEEK nanocomposite filaments. The melting point increases, and the crystallization temperature reduces as the loading level of the bioceramic particles increases, which can be ascribed to the nucleating effect of doped-HA particles leading to increased crystallization [53,54]. The melting point of PEEK increases from 343.3 °C to 348.3 °C, 351.5 °C, 356.7 °C for PEEK/SrHA and 347.5 °C, 350.1 °C, 355 °C for PEEK/ZnHA containing 0, 10, 20 and 30 wt.% filler, respectively. A summary of the thermal analysis data is given in Table 3. In addition to their nucleating effect, the presence of the bioceramic phase in the PEEK matrix hinders polymer chain mobility and increases melt viscosity [55]. This resulted in the need to optimize the extrusion and 3D-printing parameters for the doped HA loaded samples.

Figure 3b shows the crystallization behaviour of PEEK and its nanocomposite filaments during the cooling cycle. The incorporation of both Zn and Sr doped HA increases the crystallinity of PEEK by facilitating heterogenous nucleation sites for crystallization [55]. In this study, the crystallization temperature (T_c_) is decreased as the weight percent of bioceramic in PEEK is increased which shows that the crystallization is promoted in the presence of doped-HA. For pure PEEK, crystallization starts at approximately 285 °C and it is suppressed in the presence of bioceramic particles. For example, it reduces with bioceramic percentages 10, 20, 30 wt.% to 283.3 °C, 277.5 °C, 275.8 °C for SrHA and 279.4 °C, 278.2 °C, 276.2 °C for ZnHA, respectively. A summary of the thermal properties is given in Table 3.

### 3.2. 3D-Printed Sample Characterization

#### 3.2.1. X-ray Diffraction (XRD)

**Figure 4:**
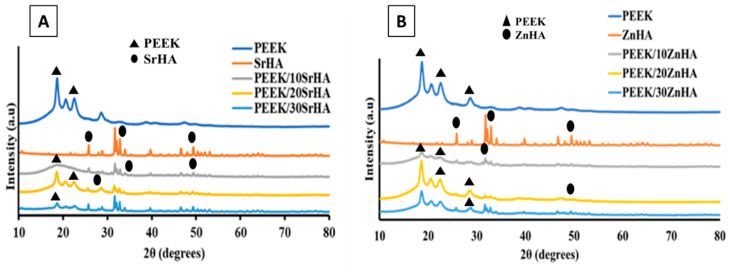
XRD analysis of PEEK and its nanocomposites with (**A**) Strontium doped hydroxyapatite (SrHA) and (**B**) Zinc doped hydroxyapatite (ZnHA), produced via 3D printing.

The crystalline phases and related characteristic peaks of PEEK, SrHA and ZnHA powders, as well as of the PEEK nanocomposite 3D-printed samples were observed by X-ray diffraction. The XRD results for the 3D-printed PEEK/doped HA samples are illustrated in Figure 4. The characteristic peaks of SrHA and ZnHA confirm the presence of bioceramics in the PEEK nanocomposites. The major diffraction peaks of PEEK were detected at approximately 19.1°, 21.3° and 22.8° [56], while the major peaks of HA were detected at approximately 31.6°, 32.8° and 49.5° [57,58,59], with slight variations due to the presence of Sr and Zn doping elements [43,59]. In the PEEK nanocomposite samples, the intensities of SrHA and ZnHA peaks gradually increase, while the intensities of peaks representing PEEK gradually decrease as the bioceramic content increases from 10 to 30 wt.% [40].

#### 3.2.2. Scanning Electron Microscopy (SEM)

**Figure 5:**
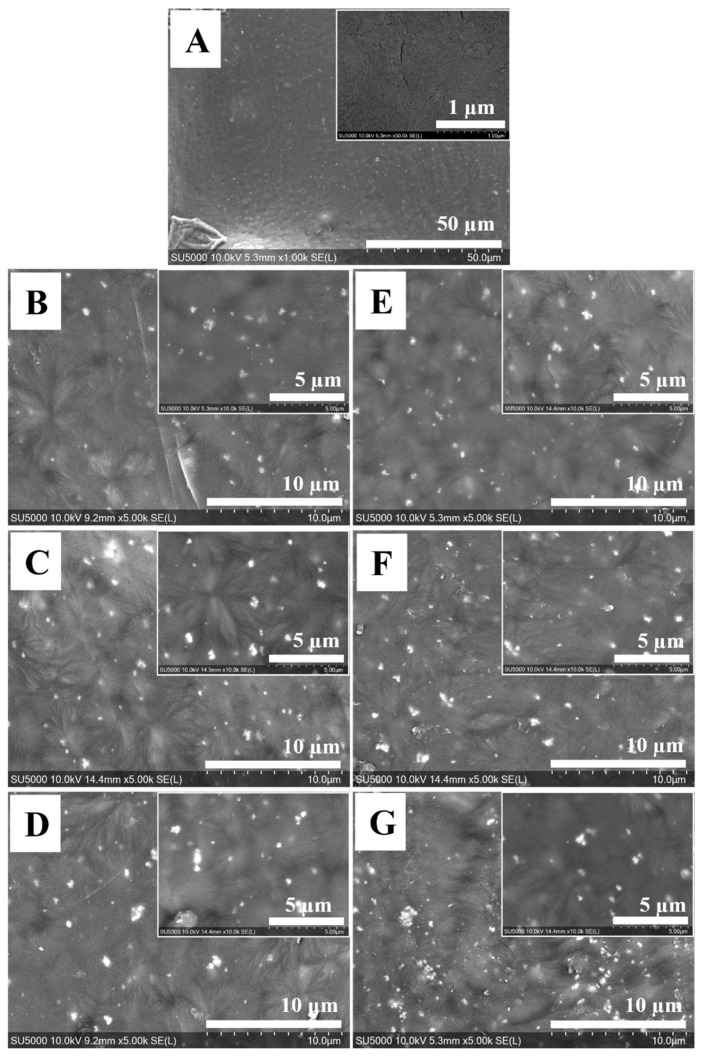
SEM images of top view of 3D-printed samples showing the distribution of bioceramic particles, tiny white dots represent the bioceramic particles, (**A**) PEEK, (**B**) PEEK/10SrHA, (**C**) PEEK/20SrHA, (**D**) PEEK/30SrHA, (**E**) PEEK/10ZnHA, (**F**) PEEK/20ZnHA, (**G**) PEEK/30ZnHA.

The top surface morphology of the 3D-printed samples was evaluated via SEM as shown in Figure 5. The doped HA particles on the surface can act as bioactive sites for apatite formation and for other biological integration activities [20,21,60]. Moreover, the addition of HA renders the PEEK surface more hydrophilic, which could help cells to attach to the surface of an implant and hence promote bone growth [18,61]. The SEM images of the top surfaces show the uniform distribution and good dispersion of the bioceramic particles in the 3D-printed PEEK samples. Figure 5A shows the surface of 3D-printed PEEK at lower and higher magnifications. As can be seen there are no bioceramic particles present in pure PEEK, as expected. In later Figure 5B–G, small white dots represent the bioceramic particles. The amount of bioceramic particles is lowest in Figure 5B,E as they contain only 10 wt.% of SrHA and ZnHA, respectively. Additionally, the distribution of the particles is uniform without any notable agglomeration. Figure 5C,F show samples with 20 wt.% of SrHA and ZnHA, respectively, in which a higher concentration of bioceramic particles is seen without significant agglomeration. Similarly, white particles increased further with few agglomerates in Figure 5D,G as they contain 30 wt.% of SrHA and ZnHA particles, respectively. Hence, it can be concluded here that up to 20 wt.%, the bioceramic particles are uniformly distributed without significant agglomeration. However, agglomerates are formed as the wt.% increases to 30 wt.%.

#### 3.2.3. Water Contact Angle Measurement

**Figure 6:**
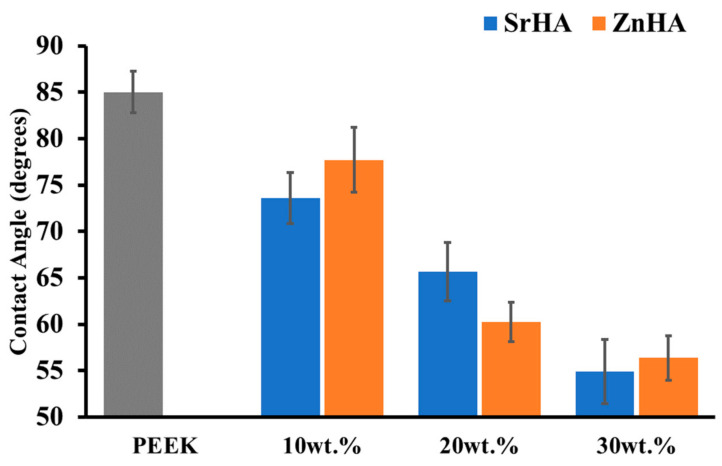
Water contact angle on the 3D-printed surfaces of PEEK and its nanocomposites with Strontium doped hydroxyapatite (SrHA) and Zinc doped hydroxyapatite (ZnHA).

Osseointegration on the surface of a material can be predicted by its wettability [62]. Together with surface porosity, surface roughness and the presence of functional groups, hydrophilicity plays a key role in the interaction with biological molecules. A hydrophilic surface is favourable for basic cell interaction mechanisms such as cell attachment and proliferation [53]. The surface of the pure PEEK polymer is hydrophobic and hence not directly favourable for cell attachment. The hydrophilicity of the PEEK surface is improved in the presence of bioceramic particles, particularly with an increase in the weight percentage of bioceramics. The contact angle reported in the literature for pure PEEK is between 70° and 90° [63,64]. As shown in Figure 6, the contact angle for pure PEEK was measured at 85.0° ± 2.2. The angle slightly decreased to 73.6° ± 2.8 for PEEK/10SrHA and 77.7° ± 3.5 for PEEK/10ZnHA. A further decrease in contact angle was detected with 20 wt.% bioceramic particles. The lowest values were observed for PEEK/30SrHA and PEEK/30ZnHA which were 54.9° ± 3.4° and 56.3° ± 2.4°, respectively. Hence, the presence of bioceramic particles increased the hydrophilicity of the surface, which is likely to result in improved osteointegration. [63,64]. The results in the form of pictures have been reported in Figure S5 (please see supplementary information).

**Figure 7:**
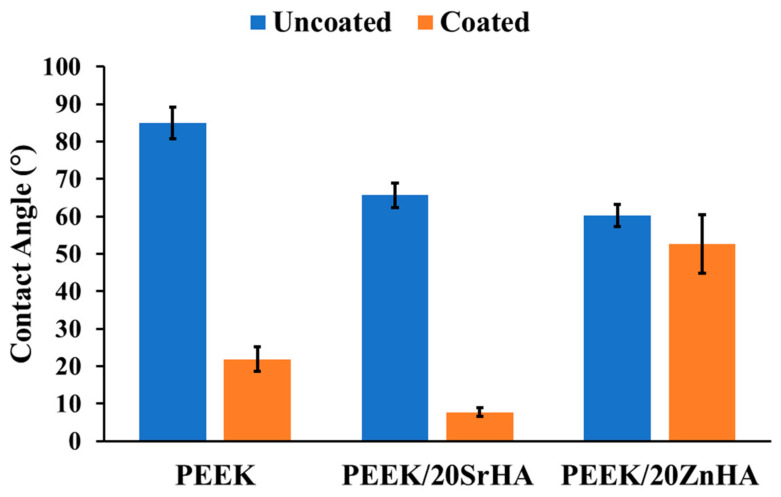
Water contact angle measurement after coating of Polyethylene glycol-DOPA (PEG_1000_-DOPA) on PEEK with 20 wt. % of Strontium doped hydroxyapatite (PEEK/20SrHA) and Zinc doped hydroxyapatite (PEEK/20ZnHA).

The water contact angle of the PEEK control and 20 wt.% samples after coating with PEG_1000_-DOPA is reported in Figure 7. The coating on the PEEK and PEEK/20SrHA samples significantly improved their surface hydrophilicity. However, only a small change in contact angle was measured for the PEEK/20ZnHA sample. This could be due to the nature of ZnHA which may have hindered the PEG_1000_-DOPA coating on its surface. However, further characterization is needed to fully investigate the effect of the coating on other properties. The improvement in the hydrophilicity of the samples after the PEG_1000_-DOPA coating can be beneficial for increasing the biocompatibility of craniomaxillofacial implants.

#### 3.2.4. Bioactivity Testing

**Figure 8:**
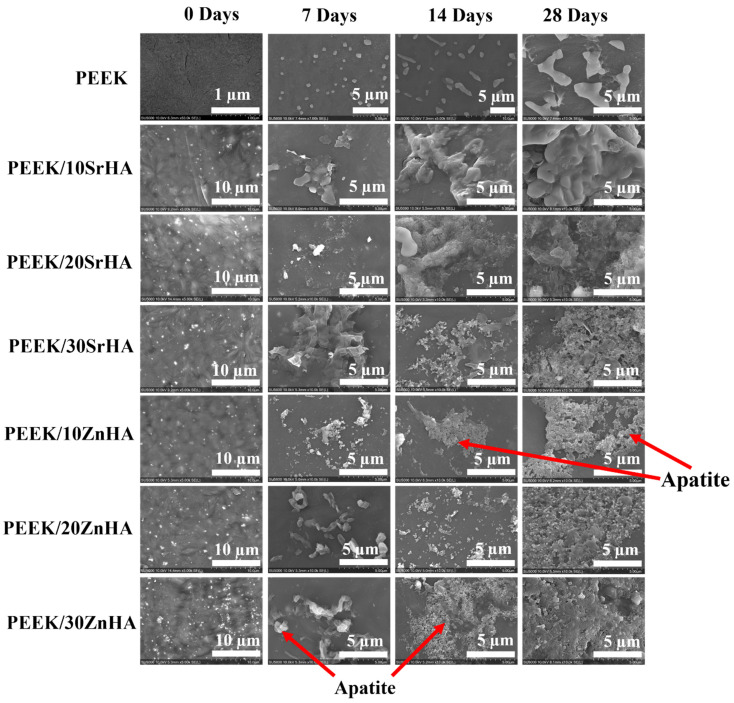
SEM images showing the apatite layer formation on the samples of PEEK and its nanocomposites with Strontium doped hydroxyapatite (SrHA) and Zinc doped hydroxyapatite (ZnHA) after immersion in SBF for 0, 7, 14, and 28 days.

The bioactivity of the samples was assessed using a simulated body fluid (SBF) immersion test. The SBF immersed samples were characterized for apatite layer formation by SEM and XRD. SEM confirmed the characteristic morphology of apatite, while XRD showed the apatite phase formation. Figure 8 shows SEM images of the sample surfaces after immersion in SBF for 0, 7, 14 and 28 days. The SBF is a super-saturated solution with ion concentrations and pH approximately equal to human blood plasma [52] and it can be precipitated out on the surface by variations in a number of factors such as pH, temperature and Ca ion concentration [65]. Thus, in order to avoid the precipitation and establishment of dynamic equilibrium, the SBF solution was replaced with fresh solution after every 48 h. There was no apatite formation observed on the surface of pure PEEK even after 28 days, which confirmed its bioinert behaviour [18]. However, apatite formation started after only 7 days of immersion on the surfaces of PEEK nanocomposites. The degree of apatite formation depends on the amount of bioceramic loading and the immersion time. The lowest apatite formation was observed for PEEK/10SrHA and PEEK/10ZnHA nanocomposites after 7 days of immersion, while the maximum apatite layer was observed for PEEK/30SrHA and PEEK/30ZnHA nanocomposites after 28 days of immersion. It is proposed that the bioceramic particles on the surface of PEEK act as bioactive sites, and higher amounts of calcium and phosphate ions are captured by these bioactive sites from the SBF solution at higher HA loadings [20]. Moreover, a longer immersion time allows higher amounts of calcium and phosphate ions to deposit on the surface. In comparison to our previous study of the bioactivity of PEEK/HA, both Sr and Zn doped HA show enhanced apatite formation in respect to pure HA [41]. Hence, incorporation of Zn and Sr doped HA is more effective for developing bioactive PEEK/HA cranial implants [57].

**Figure 9:**
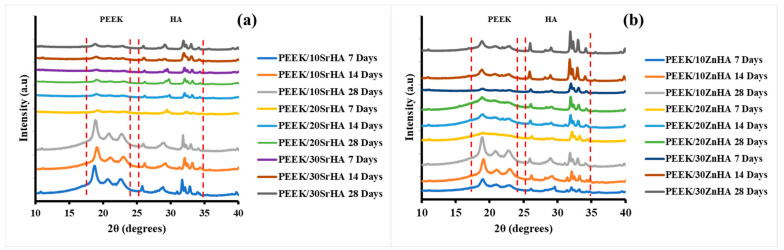
XRD of PEEK composites after immersion in SBF for 7, 14 and 28 days, PEEK with (**a**) Strontium doped hydroxyapatite (SrHA), (**b**) Zinc doped hydroxyapatite (ZnHA).

The formation of apatite on the surfaces was also confirmed by XRD graphs. Figure 9 shows the XRD analysis which was performed on PEEK/SrHA (Figure 9a) and PEEK/ZnHA (Figure 9b) after immersion in SBF for 7, 14, and 28 days. It can be seen that after immersion in SBF, no new phase was formed. The major peaks of PEEK polymer can be seen at 19.1°, 21.3° and 22.8° [56] which remain nearly same within the same composite. On the other hand, the peaks of apatite vary with the immersion time. The major peaks of apatite appeared at 31.6°, 32.8° and 49.5° [57,58,59], which slightly increased as the immersion time in SBF increased from 7 to 28 days. This confirms that as the immersion time of the samples increases in SBF, the apatite deposition also increases [66]. In Figure 9a,b with 10 wt.% of SrHA and ZnHA, respectively, the peaks in the apatite region (shown by dotted lines) slightly increased as the immersion time increased from 7 days to 28 days. However, the peaks of the PEEK region remained almost the same. A similar trend can be seen with 20 wt.% and 30 wt.% of SrHA and ZnHA. Hence, these XRD results showed that all composites formed an apatite layer on their surfaces in SBF solution which was also confirmed by SEM results.

**Figure 10:**
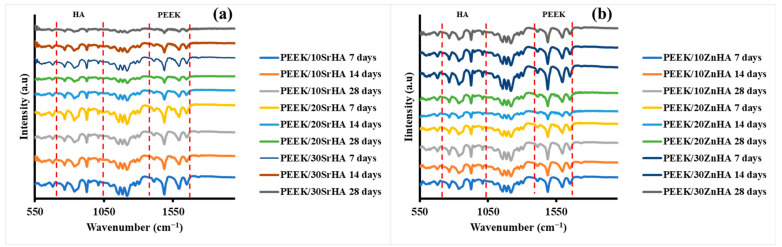
FTIR spectra of 3D printed PEEK composites with (**a**) Strontium doped hydroxyapatite (SrHA) (**b**) Zinc doped hydroxyapatite (ZnHA), immersed in simulated body fluid (SBF) for 7, 14 and 28 days.

The formation of apatite on the surfaces of 3D-printed PEEK composites has also been observed via FTIR and the spectra have been shown in Figure 10a,b. It was observed that the major peaks associated with the stretching vibration of the P-O bond due to the doped-HA appeared at 1200 cm^−1^ and 900 cm^−1^, while the carbonyl stretching vibration peak due to PEEK polymer was observed at 1655 cm^−1^ (the regions have been marked with dotted lines). The variation in the peak intensity of P-O bond was observed after immersing in SBF for 7, 14 and 28 days [67,68]. The intensity of the peaks in the apatite region within the same composite increased from 7 to 28 days for PEEK/10SrHA and PEEK/10ZnHA composites while the intensity of carbonyl of PEEK polymer decreased which could be due to the formation of apatite on the surface of the samples. Similarly, composites containing 20 and 30 wt.% of doped-HA particles showed the same trend and the FTIR results are aligned with the results observed in SEM and XRD analyses after SBF immersion.

#### 3.2.5. Mechanical Performance before SBF Immersion

**Figure 11:**
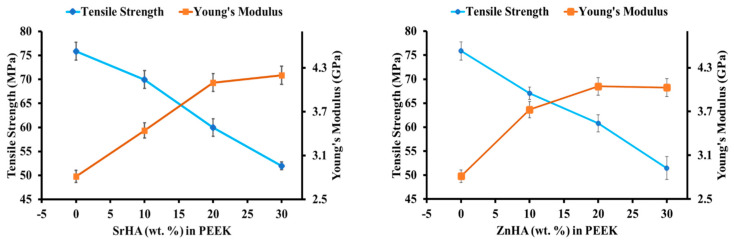
The effect of bioceramic particles on tensile strength and Young’s modulus of PEEK and its nanocomposites with Strontium doped hydroxyapatite (SrHA) and Zinc doped hydroxyapatite (ZnHA).

Results for tensile strength and Young’s modulus of PEEK and its nanocomposites, before immersion in SBF, are shown in Figure 11. As seen in Figure 11, the tensile strength decreases while the Young’s modulus increases as the amount of bioceramic increases from 0 to 30 wt.% [18,39,40]. The reduction in tensile strength with the addition of bioceramic may be due to weak interfacial adhesion and a mismatch of stiffness between the particles and the polymer matrix which can induce stresses and weak points within the nanocomposites [69]. When a load is applied, the poor interfacial bond can result in premature crack formation and rupture at lower levels of stress [40,69,70]. This phenomenon was more prominent at higher loading levels. In this study, the tensile strength of PEEK was measured at 75.1 MPa. In comparison with pure PEEK, the tensile strength decreased approximately by 7.9%, 25.0%, 31.5% with the addition of 10, 20, 30 wt.% of SrHA, respectively. Similarly, the tensile strength decreased approximately by 11.8%, 19.7%, 32.3% with the addition of 10, 20, 30 wt.% ZnHA, respectively. The mechanical strength of 30 wt.% nanocomposites decreased more which could be due to the presence of agglomerates, as discussed in Section 3.2.2. On the other hand, the Young’s modulus increased with the addition of SrHA and ZnHA to the PEEK matrix. Hence, the changes in the mechanical properties were more dependent on the doped HA content compared to the nature of the doping elements. The tensile strength and Young’s modulus of cortical bone is in the range of 50–150 MPa [50] and 2–8 GPa [71], respectively. Hence, the samples produced by 3D printing in this study have adequate strength and a modulus suitable for orthopaedic implants. However, the samples containing 10 wt.% and 20 wt.% ZnHA and SrHA can be considered as better options in this respect [18,39,40].

#### 3.2.6. Mechanical Performance after SBF Immersion

**Figure 12:**
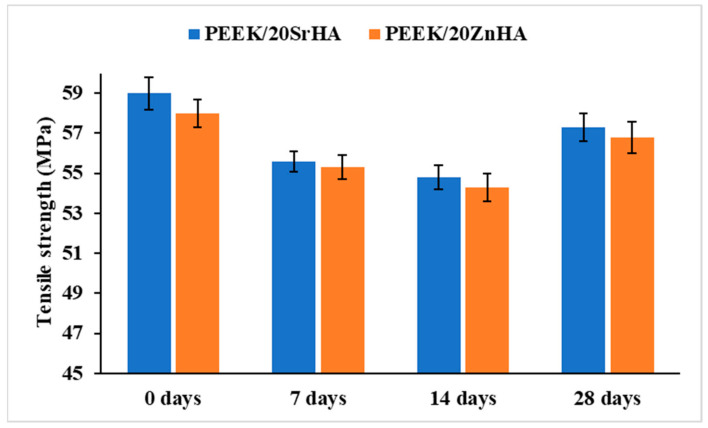
Tensile testing results of samples containing PEEK with Strontium doped hydroxyapatite (SrHA) and Zinc doped hydroxyapatite (ZnHA), 20 wt.% of bioceramic particles after immersion in SBF for 7, 14, 28 days.

The mechanical test results after immersion in SBF are shown in Figure 12. It can be observed that the tensile strength decreases slightly after 7 days and 14 days of immersion in SBF by 5.9% and 8.4% for SrHA and 4.9% and 6.7% for ZnHA, respectively. However, no further decrease in tensile strength is observed after 28 days of immersion in SBF. The slight decrease in strength after 7 and 14 days of immersion could be due to the formation of a metastable phase of calcium hydrogen phosphate which can form due to an interaction between bioceramic particles and the ions [Ca^+2^ and (PO_4_)^−3^] in the SBF solution [20] or due to the dissolution of doped HA in the solution leaving behind voids which act as defects during load bearing [72,73,74]. It is anticipated that, as the immersion time increases to 28 days, the metastable phase converts into stable bone-like apatite and covers the whole surface as a thin layer [20]. Hence, the formation of a thin apatite layer could be the reason for the slightly improved tensile strength of the samples after 28 days of immersion. This also suggests that after implantation, the chance of mechanical failure of the implant is higher before 14 days and therefore more care should be taken. Long-term immersion studies are required in the future to investigate the effect of apatite layer formation on the mechanical performance of PEEK nanocomposites. This study provides an insight into what can potentially occur when bioactive PEEK implants are inserted in the body at the initial stages.

#### 3.2.7. Impact Testing

**Figure 13:**
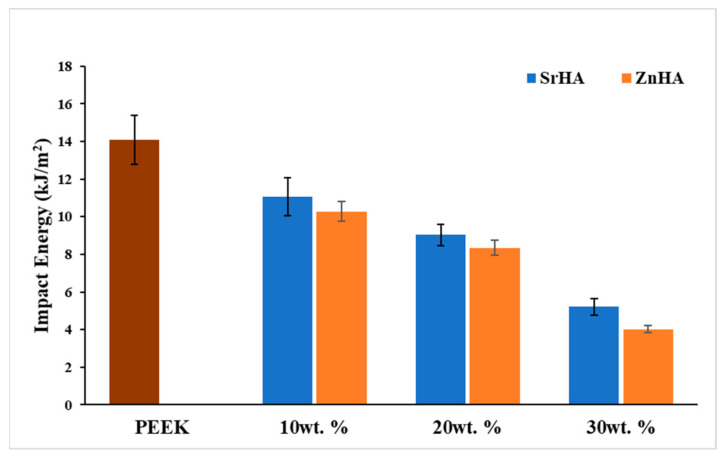
Izod impact strength of PEEK and its nanocomposites with Strontium doped hydroxyapatite (SrHA) and Zinc doped hydroxyapatite (ZnHA) at different weight percentages measured from unnotched samples according to ISO 180:2000.

The impact strength of PEEK and its nanocomposites is shown in Figure 13. The impact strength of PEEK is measured as 14.1 kJ/m^2^ and it decreases as the percentage of bioceramic particles increases. This may be an indication of weak bonding between the bioceramic particles and the PEEK matrix, resulting in crack initiation and propagation in the region of impact. Hence, samples containing ceramic particles absorbed less energy before a break as compared to pure PEEK. This phenomenon was more prominent as the weight percentage of the bioceramic increased. In this study, by incorporating 10, 20 and 30 wt.% bioceramic, the impact strength was measured as 11.1 kJ/m^2^, 9.0 kJ/m^2^, 5.2 kJ/m^2^ for SrHA samples and 10.3 kJ/m^2^, 8.4 kJ/m^2^, 4.0 kJ/m^2^ for ZnHA samples, respectively. The amount of energy absorbed by the skull bone is reported in the range of 3 to 9 kJ/m^2^ and depends on the age of the patient, type of cranial bone as well as the loading rate [75]. In this study, the measured values for PEEK and its nanocomposites fall within that range.

## 4. Conclusions

In this study, PEEK and its nanocomposites with SrHA and ZnHA up to 30 wt.% have been processed by novel route i.e., FDM 3D printing, and the parameters for filament extrusion and 3D printing processes were optimized and reported for the first time. Micro-CT and SEM analysis suggested a uniform distribution of bioceramic particles in PEEK. Three-dimensional-printed samples were successfully fabricated via FDM and characterized via XRD, SEM, DSC, water contact angle, tensile testing before and after SBF immersion and impact testing. XRD analysis confirmed the presence of doped HA in PEEK. SEM showed that the bioceramic particles were uniformly distributed on the surfaces and played a role in the bioactivity of the samples in SBF. DSC results showed the melting points and crystallinity of nanocomposites increased by the addition of bioceramic particles up to 30 wt.% from 343 °C to 355 °C and 27.7% to 34.6%, respectively. In the presence of bioceramic particles, the surface hydrophilicity of nanocomposites, as indicated by water contact angle values was considerably improved from 85° to 55° which further decreased to 10° after coating with PEG_1000_-DOPA. Moreover, it was observed that the tensile and impact strength of PEEK decreased from 75 MPa to 51 MPa with the addition of SrHA and ZnHA up to 30 wt.%, which could be attributed to the weak attachment between PEEK and doped-HA and the brittle nature of bioceramic particles. However, the elastic modulus and bioactivity -as indicated by SBF immersion studies- increased with the addition of bioceramic particles, due to the stiffness and bioactive nature of the particles, respectively. Samples containing 20 wt.% bioceramic were selected for tensile testing after SBF immersion. The tensile strength declined slightly from 59 MPa to 55 MPa after SBF immersion of up to 14 days and then increased slightly to 57.5 MPa after 28 days immersion in SBF, which could be due to the formation of an apatite layer over two weeks. In addition, impact strength decreased from 14 kJ/m^2^ to 4 kJ/m^2^ by the addition of bioceramic particles up to 30 wt.% and in the range of impact strength of human skull bone. Overall, the study showed that PEEK/SrHA and PEEK/ZnHA nanocomposites can be successfully processed via FDM, and the resulting 3D-printed nanocomposites have great potential for use in manufacturing craniomaxillofacial implants due to their mechanical performance and increased bioactivity.

## Figures and Tables

**Table 1:** Optimised parameters for preparing 1.75 ± 0.05 mm diameter filaments for PEEK and its nanocomposites using a desktop extruder (3devo Composer 450). Different zone temperatures (Zone 1; Z1, Zone 2; Z2, Zone 3; Z3, Zone 4; Z4) in the extruder for PEEK with Strontium doped hydroxyapatite (PEEK/SrHA) and with Zinc doped hydroxyapatite (PEEK/ZnHA) have been reported.

Parameters/ Material	PEEK	PEEK/10SrHA & PEEK/10ZnHA	PEEK/20SrHA & PEEK/20ZnHA	PEEK/30SrHA & PEEK/30ZnHA
Temperature (°C) Z_1_, Z_2_, Z_3_, Z_4_	355, 365, 375, 390	360, 370, 380, 400	365, 380, 400, 410	380, 400, 410, 420
Feeding rate (g/min)	3.3	2.8	2.3	2.1
Extruder Screw speed (RPM)	5.5	5.5	5.5	5.5
Cooling fan speed (%)	100	90	80	80
Nozzle diameter (mm)	4	4	4	4
Puller wheel speed (RPM)	1100	1000	950	900

**Table 2:** Optimised parameters reported for 3D printing of PEEK and its nanocomposites with Strontium doped hydroxyapatite (SrHA) and Zinc doped hydroxyapatite (ZnHA).

	PEEK	PEEK/10SrHA & PEEK/10ZnHA	PEEK/20SrHA & PEEK/20ZnHA	PEEK/30SrHA & PEEK/30ZnHA
Nozzle temperature (°C)	390	410	420	430
Bed temperature (°C)	150	160	180	200
Chamber temperature (°C)	75	80	80	80
Layer thickness (mm)	0.2	0.2	0.2	0.2
Printing speed (mm/s)	30	30	30	30
Infill density (%)	100	100	100	100
Infill pattern	−45, +45	−45, +45	−45, +45	−45, +45
Nozzle diameter (mm)	0.5	0.5	0.5	0.5

**Table 3:** Thermal properties of PEEK and its nanocomposite filaments by DSC.

	T_g_, Mid-Point (°C)	T_m_ (°C)	T_c_ (°C)	Xc (%)
PEEK	143.3	343.1	285.8	27.7
PEEK/10SrHA	144.1	348.3	283.3	28.1
PEEK/10ZnHA	143.2	347.5	279.4	28.7
PEEK/20SrHA	146.7	351.5	277.5	30.5
PEEK/20ZnHA	144.4	350.1	282.2	31.5
PEEK/30SrHA	148.1	356.7	276.8	32.3
PEEK/30ZnHA	145.3	355.0	277.2	34.6

## Data Availability

The raw/processed data required to reproduce these findings are available from the authors on request.

## Supplementary Materials

The following supporting information can be downloaded at: https://www.mdpi.com/article/10.3390/polym14071376/s1, Figure S1: Non-uniform diameter of PEEK nanocomposite filaments obtained during optimisation via 3devo desktop extruder; Figure S2: PEEK composite filaments prepared after optimisation via 3devo desktop extruder; (a) PEEK, (b) PEEK/10SrHA, (c) PEEK/20SrHA, (d) PEEK/30SrHA; Figure S3: FDM 3D printing optimisation of PEEK nanocomposites at (a) low nozzle temperature, (b) low printing speed (c) high printing speed, (d) low bed and chamber temperatures, (e,f) successful prints of impact testing samples; Figure S4: Tensile testing samples of PEEK nanocomposites prepared at optimised conditions via SpiderBot FDM 3D printer; Figure S5: Water contact angle on the surface of 3D printed samples; (a) PEEK, (b) PEEK/10SrHA, (c) PEEK/20SrHA, (d) PEEK/30SrHA, (e) PEEK/10ZnHA, (f) PEEK/20ZnHA, (g) PEEK/30ZnHA.
