# Supplementary material for: 3D Printed Strontium and Zinc Doped Hydroxyapatite Loaded PEEK for Craniomaxillofacial Implants
Source: Polymers (Basel). 2022 Mar 28;14(7):1376. doi: 10.3390/polym14071376 (PMC9002955; doi:10.3390/polym14071376)
Supplement: Supplementary file 1 [file polymers-14-01376-s001.zip › polymers-1606035-supplementary.pdf]

polymers

MDPI

Supplementary Information

3D Printed Strontium and Zinc Doped Hydroxyapatite Loaded PEEK for Craniomaxillofacial Implants

Faisal Manzoor [1,*], Atefeh Golbang [1,*], Dorian Dixon [2], Elena Mancuso [2], Usaid Azhar [3,4], Ioannis Manolakis [3,4], Daniel Crawford [5], Alistair McIlhagger [1] and Eileen Harkin-Jones [1]

[1] Department of Mechanical Engineering, School of Engineering, Ulster University, Shore Road, Newtownabbey BT37 0QB, UK; a.mcilhagger@ulster.ac.uk (A.M.); e.harkin-jones@ulster.ac.uk (E.H.-J.)
[2] Nanotechnology and Integrated Bio-Engineering Centre (NIBEC), Ulster University, Shore Road, Newtownabbey BT37 0QB, UK; d.dixon@ulster.ac.uk (D.D.); e.mancuso@ulster.ac.uk (E.M.)
[3] Precision Engineering, Materials & Manufacturing (PEM) Research Centre, Institute of Technology Sligo, Ash Lane, F91 YW50 Sligo, Ireland; usaid.azhara@mail.itsligo.ie (U.A.); manolakis.ioannis@itsligo.ie (I.M.)
[4] Department of Life Sciences, Institute of Technology Sligo, Ash Lane, F91 YW50 Sligo, Ireland
[5] Axial 3D, Alexander House, 17a Ormeau Ave, Belfast BT2 8HD, UK; d.crawford@axial3d.com
* Correspondence: manzoor-f@ulster.ac.uk (F.M.); a.golbang@ulster.ac.uk (A.G.)

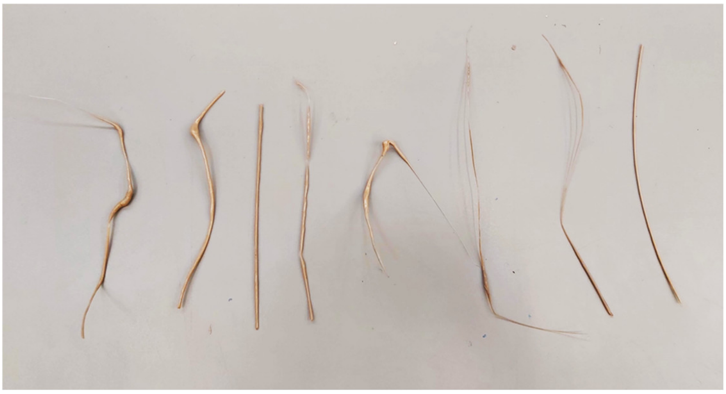

Figure S1. Non-uniform diameter of PEEK nanocomposite filaments obtained during optimisation via 3devo desktop extruder.

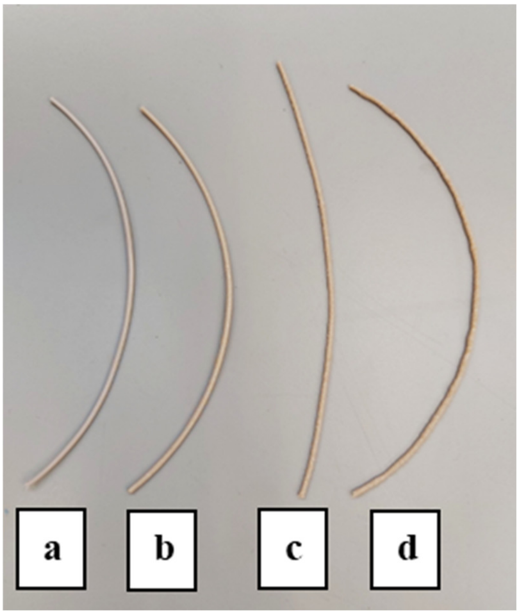

Figure S2. PEEK nanocomposite filaments prepared after optimisation via 3devo desktop extruder; (**a**) PEEK, (**b**) PEEK/10SrHA, (**c**) PEEK/20SrHA, (**d**) PEEK/30SrHA.

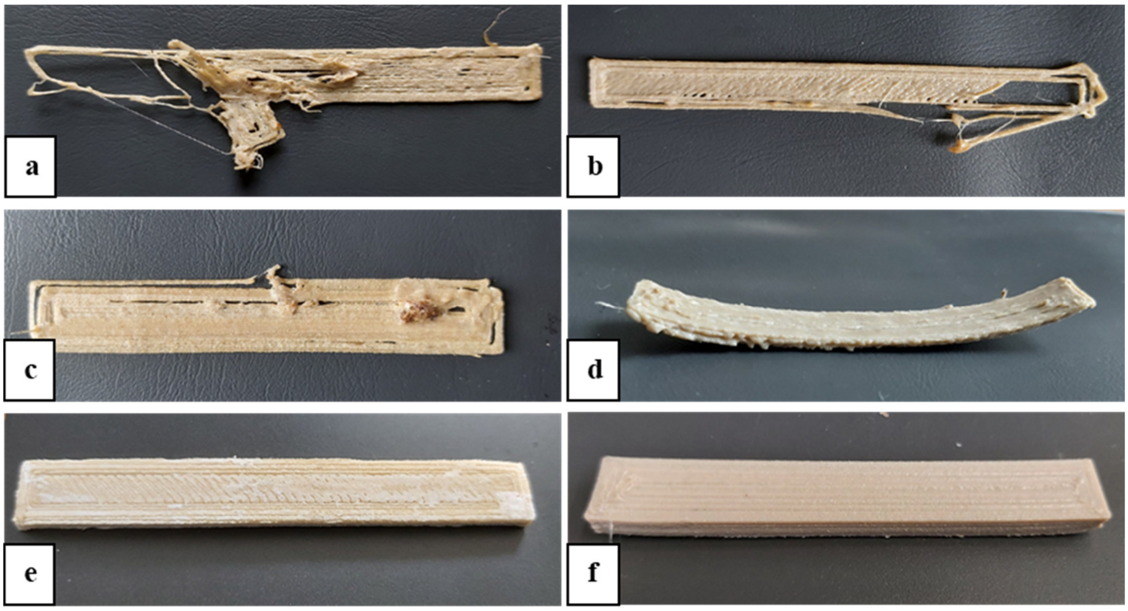

Figure S3. FDM 3D printing optimisation of PEEK nanocomposites at (**a**) low nozzle temperature, (**b**) low printing speed (**c**) high printing speed, low bed and chamber temperatures, (**e**,**f**) successful prints of impact testing samples.

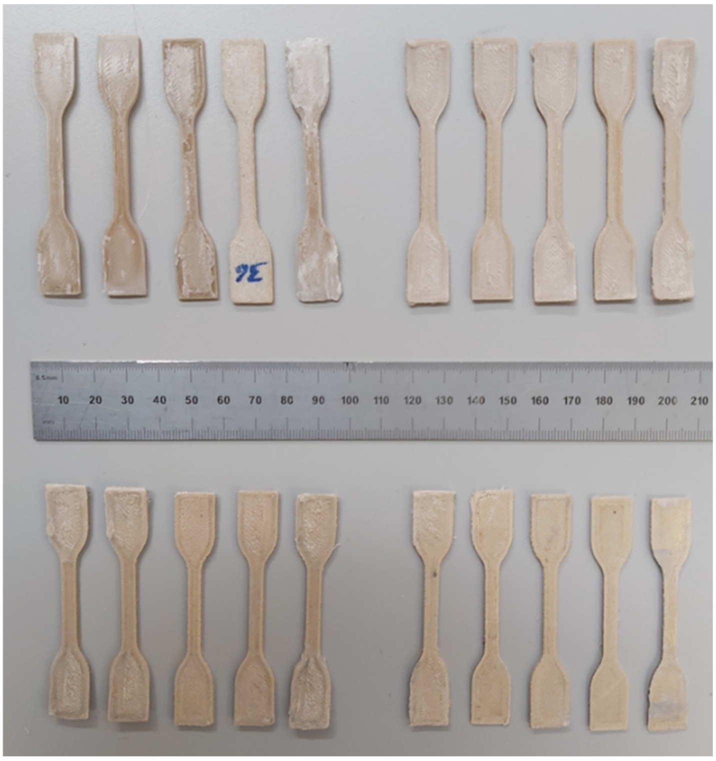

Figure S4. Tensile testing samples of PEEK nanocomposites prepared at optimised conditions via SpiderBot FDM 3D printer.

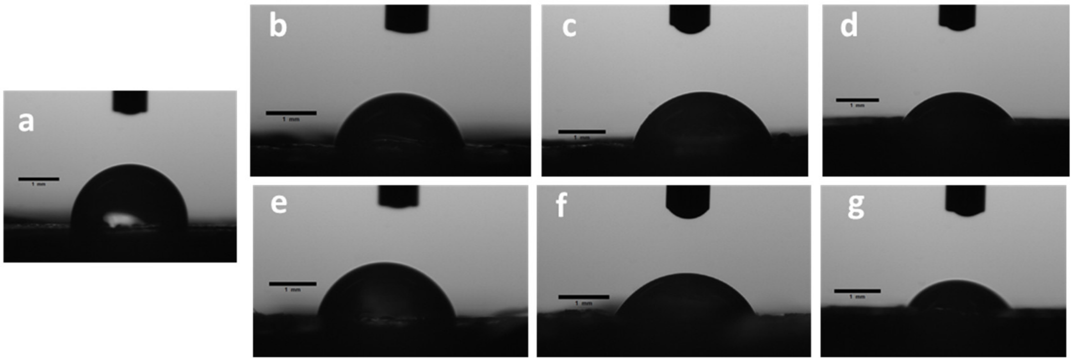

Figure S5. Water contact angle on the surface of 3D printed samples; (**a**) PEEK, (**b**) PEEK/10SrHA, (**c**) PEEK/20SrHA, (**d**) PEEK/30SrHA, (**e**) PEEK/10ZnHA, (**f**) PEEK/20ZnHA, (**g**) PEEK/30ZnHA.
